# Ruptured Coronary Sinus of Valsalva in the Setting of a Supracristal Ventricular Septal Defect

**DOI:** 10.5811/cpcem.2019.11.44008

**Published:** 2020-02-24

**Authors:** Abilio Arrascaeta-Llanes, Akanksha Kashyap, Diana Meyler, Ravi Gupta, Zubin Tharayil, Waqas Khan

**Affiliations:** * Long Island Community Hospital, Department of Medicine, Patchogue, New York; † Long Island Community Hospital, Department of Cardiology, Patchogue, New York

## Abstract

A sinus of Valsalva aneurysm (SOVA) is usually a silent entity until one of its complications arises, such as heart failure. SOVA itself is uncommon, but it is more frequently associated with a supracristal ventricular septal defect (SVSD). We present a 67-year-old man with a history of an asymptomatic SVSD who presented to the emergency department with signs and symptoms of heart failure. He was subsequently found to have a ruptured SOVA and underwent urgent surgical repair.

## INTRODUCTION

Clinical presentations consistent with heart failure, conduction abnormalities, and acute coronary syndrome are frequently encountered in the emergency department (ED). A patient with a sinus of valsalva aneurysm (SOVA) may present with one of the above manifestations. SOVA is a rare cardiac condition usually resulting from a congenital incomplete fusion of the aortic media and the aortic valve annulus. It can also be caused by processes that compromise the septal wall, such as supracristal ventricular septal defects (SVSD), endocarditis, syphilis, cystic medial necrosis, and chest trauma. If ruptured, SOVA presents with symptoms of a large left-to-right shunt causing severe heart failure, necessitating urgent surgical intervention.

## CASE REPORT

A 67-year-old Caucasian male presented to the ED with a three-day history of worsening dyspnea, cough, chills, and body aches. He denied chest pain, fever, recent travel, leg swelling, sick contacts, and syncope. His past medical history, prior to his ED presentation, included a ventricular septal defect (VSD), chronic atrial fibrillation, left ventricular hypertrophy, hypertension, and dyslipidemia; he denied any surgical history. During his last visit with his cardiologist six months prior to presentation, the VSD was stable, and he was asymptomatic. In the ED, his blood pressure was 131/60 millimeters of Mercury (mmHg), heart rate was 102 beats per minute, respiratory rate was 27 breaths per minute and arterial oxygen saturation was 95% on two liters of supplemental oxygen. The remainder of the physical exam was significant for end-expiratory wheezing, an irregularly irregular pulse, and a loud, precordial continuous systolic murmur with right-sided prominence; no cyanosis, clubbing or lower extremity edema was noted. Laboratory results were significant for an elevated troponin-I of 0.063 nanogram/milliliter (ng/ml) (normal range: 0.0–0.045 ng/ml) and brain natriuretic peptide of 744 picogram/milliliter (pg/ml) (100–400 pg/ml); a complete blood count and a basic metabolic panel were within normal limits. An electrocardiogram revealed atrial fibrillation with left ventricular hypertrophy and a chest radiograph was unremarkable. The patient was admitted for presumed bronchitis and diastolic congestive heart failure exacerbation and subsequently treated with diuretics, bronchodilators, and antibiotics.

**Image 1:**
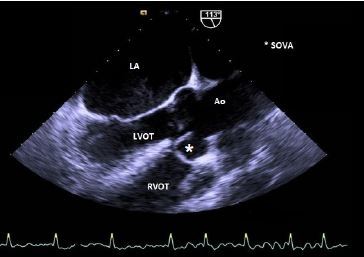
Transesophageal echocardiogram demonstrating an eccentrically aneurismal right sinus of Valsalva (star). *LA*, left atrium*; LVOT*, left ventricle outflow tract; *Ao*, aorta; *RVOT*, right ventricle outflow tract; *SOVA*, sinus of valsalva aneurysm.

**Image 2:**
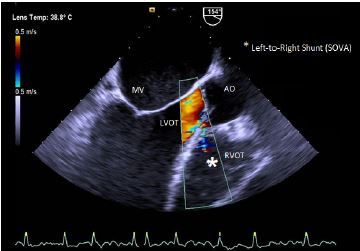
Tranesophageal echocardiogram demonstrating a ruptured sinus of valsalva aneurysm (star). *MV*, mitral valve; *Ao*, aorta; *RVOT*, right ventricle outflow tract; *LVOT*, Left ventricle outflow tract.

Despite the aforementioned treatment, the patient did not clinically improve. The following day, a transthoracic echocardiogram (TTE) revealed a small-to-moderate sized supracristal VSD with a left-to-right shunt. The TTE also showed a moderately dilated left ventricle cavity with a left ventricular ejection fraction (LVEF) of 50%, bilateral atrial and aortic enlargement and a severely elevated resting pulmonary artery (PA) pressure of 55 mmHg with preserved contractility of the right ventricle. A subsequent transesophageal echocardiogram (TEE) showed a mild-to-moderately dilated aortic root at the level of the sinuses of Valsalva, with normal aortic valve annular dimensions. The right coronary sinus of Valsalva was eccentrically aneurysmal and ruptured, extending anteriorly and inferiorly into the right ventricle outflow tract (RVOT) near the patient’s membranous VSD, exhibiting a “windsock effect” (Image 1,2).

A high-velocity turbulent flow was visualized from the aortic root to the RVOT. There was a small restrictive supracristal membranous VSD with a minimal shunt flow. The left ventricle was globular and Left Ventricle Ejection Fraction (LVEF) was estimated to be 25%. The right ventricle function was impaired with significant pulmonary artery hypertension.

**Image 3:**
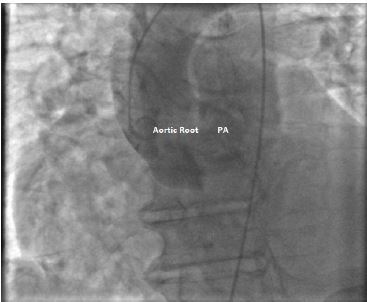
Aortic root angiogram. Intravenous contrast shows blood flowing into the pulmonary artery (PA) from the left ventricle outflow tract.

The patient underwent an urgent right-and-left sided cardiac catheterization, which confirmed the presence of a right SOVA rupture into the RVOT with blood flow into the PA on left ventricular and aortic root angiogram (Image 3). The PA was dilated with a Pulmonary-Systemic flow ratio (Qp/Qs) of 5.33 due to the left-to-right shunt. Additionally, moderate pulmonary hypertension and moderate global left ventricular dysfunction (ejection fraction 40%) were noted.

The patient was transferred to a tertiary care facility for urgent surgical repair of the acute left-to-right SOVA-RVOT shunt. During the surgery, the aortic root was accessed via a transaortic route. The right coronary sinus had a 4-millimeter tear with an aneurysmal dilation to the RVOT. The aneurysm was resected and the area was reconstructed with a bovine pericardial patch; the SVSD was also repaired with a bovine pericardial patch. The aortic valve was not involved and did not need repair. The patient performed well postoperatively and was eventually discharged.

**CPC-EM Capsule**

What do we already know about this clinical entity?

Sinus of Valsalva Aneurysm (SOVA) is a rare, often congenital, cardiac condition resulting from incomplete fusion of the aortic media and the aortic valve annulus.

What makes this presentation of disease reportable?

Our presentation involves an elderly male with history of asymptomatic supracristal ventricular septal defects and confounding cardiac history who presents with signs and symptoms of heart failure.

What is the major learning point?

Physicians should consider ruptured SOVA in the differential for patients with a history of ventricular septal defects presenting with cardiopulmonary symptoms.

How might this improve emergency medicine practice?

Awareness of the various disease manifestations of SOVA and performing an early echocardiogram can lead to early recognition and prevent delays in appropriate therapy.

## DISCUSSION

A supracristal ventricular septal defect is a subtype of VSD that results from the absence of the subpulmonary muscular infundibulum, leaving a fibrous continuity between the aortic and pulmonic valves.^1^,^2^ SVSD affects the Asian population to a greater degree than the Caucasian population (5:1). The SVSD produces a left-to-right shunt with a venturi effect that results in an acquired aortic valve deformity, including the herniation of the right aortic sinus with the possible development of an aneurysm.^3^ The prevalence of aortic valve prolapse is around 1% at 1 year of age and 70% at 15 years of age.^4^

SOVA is an aneurysmal dilatation of one or more aortic sinuses between the annulus of the aortic valve and the sinotubular junction. SOVA can be classified as congenital when there is a deficiency of lamina elastica and muscular tissue surrounding the aorta or there is the presence of an acquired weakness of the aforementioned structures.^5^,^6^ They frequently co-occur with ventricular septal defects, aortic valve dysfunction, or other cardiac abnormalities. Although unruptured ASVs are usually asymptomatic, ruptured ASVs often cause symptoms similar to those of heart failure and produce a continuous, mechanical-sounding murmur. Transsternal or transesophageal echocardiography is usually effective in detecting ASVs. Because symptomatic ASVs pose significant risks for the patient, and because the repair of asymptomatic ASVs generally produces excellent outcomes, surgery is indicated in most cases. The primary goals of surgical repair are to close the ASV securely, remove or obliterate the aneurysmal sac, and repair any associated defects. Operative mortality is generally low except in patients with concomitant bacterial endocarditis or other infections. Late events are uncommon and tend to be related to aortic valve prothesis or Marfan syndrome.”The most frequent etiologies of congenital SOVA are Marfan’s Syndrome, Ehlers-Danlos Syndrome, a bicuspid aortic valve and other connective tissue disorders. An acquired SOVA can result from degenerative connective tissue diseases, chest trauma, atherosclerosis, and infections such as syphilis, bacterial endocarditis, and tuberculosis.

The incidence of SOVA in the western population is approximately 0.09%, occurring predominantly in the male population. It represents 0.1–3.5% of all congenital heart diseases.^7^ ASVs occur much more frequently in the right coronary sinus of Valsalva. Previous reports, based on necropsy and cardiac surgery findings, estimated that 20% of ASVs are unruptured. Patients with an unruptured ASV may remain asymptomatic for a long period of time until rupture. They may also present with dyspnea, palpitation, and angina-like chest pain. Aortic insufficiency in the patients with unruptured ASVs is common, and other valvular lesions can be observed in these patients as well. Echocardiography, as a noninvasive and portable tool, is widely used to detect ASVs. Additionally, computed tomography and cardiac magnetic resonance imaging, alone or in combination, can provide precise information about its anatomic extension and intrinsic characteristics of the pathology.”^8^ Between the ages of 30 to 45 years, 80% of patients with a SOVA will become symptomatic; its detection is usually triggered by the manifestation of a SOVA complication. An unruptured SOVA can cause an obstruction of the RVOT, a conduction block, aortic regurgitation and/or a transient ischemic attack. A moderate-to-severely dilated SOVA can lead to blood stasis and predisposes to thrombus formation; thrombus occlusion of the coronary arteries can mimic an acute coronary syndrome.^9^ A ruptured SOVA typically presents with an audible continuous machine-like murmur, bounding pulses, aortic regurgitation and a left-to-right shunt leading to acute heart failure.^4^ Rupture typically occurs in 30% of the patients, with 30–40% of ruptures occurring between 20 and 40 years of age.^1^,^6^,^10^

Traditionally, TTE and TEE are the initial diagnostic tests for SOVA.^11^,^12^ Both techniques can assess for a shunt; however, they lack sensitivity in detecting shunts less than 3 millimeters. It can be technically challenging to assess for ventricular defects, as the prolapsed cusp can occlude the shunt, resulting in a missed diagnosis in 9% of patients. Prior to surgery, contrast magnetic resonance imaging and aortography are the diagnostic tests of choice in order to better characterize the shunts.^13^

For an unruptured SOVA, the timing of surgical intervention depends on the size of the aneurysm, the rate of progression, and any associated cardiac abnormalities, such as bicuspid valves or connective tissue diseases. An unruptured SOVA with any of the following characteristics requires surgery: greater than 5.5 centimeters (cm) in size; a yearly growth rate greater than 0.5 cm; 5.0 cm-5.5 cm in the presence of bicuspid valves or 4.0 cm-5.5 cm in the setting of connective tissue disease.^14^ Yearly follow up, with either cardiac computed tomography or magnetic resonance imaging, is recommended for an unruptured SOVA.

A ruptured SOVA requires urgent surgical or percutaneous correction. Percutaneous correction is increasingly favored in both elective repair of an unruptured SOVA and in urgent repair of ruptured SOVA.^15^ Surgical SOVA repair is performed via either primary closure or bovine patch; primary closure is preferred for repairing a small SOVA and patch closure is generally used for larger defects. The mortality rate associated with the surgery varies from 1.9% to 3.6% and the survival rate is approximately 90% at 15 years.^16^

## CONCLUSION

Emergency physicians should keep a ruptured SOVA in the differential for patients with a known history of ventricular septal defects who present with acute heart failure, acute coronary syndrome, conduction abnormalities or transient ischemic attacks. Performing an early echocardiogram when the clinical picture is unclear can help delineate between diagnoses and prompt further investigation into the etiology. This can increase the probability of early identification of a SOVA and facilitate an appropriate management plan.
